# Differences in amyloid PET positivity based on ethnoracial group and social determinants of health: The new IDEAS study

**DOI:** 10.1002/alz.71406

**Published:** 2026-05-27

**Authors:** Corey J. Bolton, Peggye Dilworth‐Anderson, Jon Steingrimsson, Maryanne Thangarajah, Lucy Hanna, Constantine Gatsonis, Charles Windon, Maria C. Carrillo, Emily Glavin, Ilana Gareen, Margo B. Heston, Bruce E. Hillner, Andrew March, Robert A. Rissman, Barry A. Siegel, Karen Smith, Rachel A. Whitmer, Christopher J. Weber, Gil D. Rabinovici, Consuelo H. Wilkins

**Affiliations:** ^1^ Department of Medicine Division of Geriatric Medicine Vanderbilt University Medical Center Nashville Tennessee USA; ^2^ Vanderbilt Memory and Alzheimer's Center Vanderbilt University Medical Center Nashville Tennessee USA; ^3^ Health Policy and Management Gillings School of Global Public Health University of North Carolina at Chapel Hill Chapel Hill North Carolina USA; ^4^ Department of Biostatistics Brown University Providence Rhode Island USA; ^5^ Center for Biostatistics and Health Data Science Brown University School of Public Health Providence Rhode Island USA; ^6^ Department of Neurology Edward and Pearl Fein Memory and Aging Center Weill Institute for Neurosciences University of California, San Francisco San Francisco California USA; ^7^ Alzheimer's Association Chicago Illinois USA; ^8^ Center for Research and Innovation American College of Radiology Reston Virginia USA; ^9^ Department of Epidemiology Brown University School of Public Health Providence Rhode Island USA; ^10^ Department of Medicine Virginia Commonwealth University Richmond Virginia USA; ^11^ Department of Physiology and Neuroscience Alzheimer's Therapeutic Research Institute Keck School of Medicine of the University of Southern California San Diego California USA; ^12^ Edward Mallinckrodt Institute of Radiology Washington University School of Medicine St. Louis Missouri USA; ^13^ Division of Research Kaiser Permanente Oakland California USA; ^14^ Department of Public Health Sciences University of California Davis Davis California USA; ^15^ Department of Radiology and Biomedical Imaging University of California, San Francisco San Francisco California USA

**Keywords:** Alzheimer's, amyloid PET, disparities, ethnoracial differences

## Abstract

**INTRODUCTION:**

This study leverages a large, diverse cohort to characterize ethnoracial differences in amyloid positron emission tomography (PET) positivity and identify social determinants of health (SDOHs) contributing to these differences.

**METHODS:**

We assessed differences in amyloid PET positivity by ethnoracial group (Black, Latinx, or all other races/ethnicities [AORE]) among Medicare beneficiaries with cognitive impairment. Secondary analyses associated various SDOHs with amyloid PET positivity.

**RESULTS:**

Among 5757 participants (21.7% Black, 20.3% Latinx, 58.1% AORE), we found lower odds of amyloid positivity in Black (odds ratio [OR]: 0.72, 95% confidence interval [CI]: 0.62–0.83) and Latinx (OR: 0.78, 95% CI: 0.67–0.91) compared to AORE. Individuals in the comfortable (OR: 1.22, 95% CI: 1.05–1.42) and distressed (OR: 1.40, 95% CI: 1.08–1.82) Area Deprivation Index (ADI) groups had greater odds of amyloid positivity than individuals in the prosperous group.

**DISCUSSION:**

Amyloid PET positivity rates were lower among Black and Latinx individuals and higher among individuals in more deprived ADI categories. This has potential implications for anti‐amyloid therapies.

## Highlights

- Amyloid positron emission tomography (PET) positivity in cognitively impaired adults differed by ethnoracial group.
- Black/African American & Hispanic/Latinx groups had lower amyloid positivity rates.
- Social determinants of health did not explain differing amyloid positivity rates.
- Results highlight the need for non‐amyloid interventions for cognitive decline.

## 1 BACKGROUND

Early detection of Alzheimer's disease (AD) and related dementias (ADRDs) is essential for appropriate disease management and improved patient care, and is increasingly important due to emerging therapies.^1^ Equitable access to early diagnosis is crucial as Black and Latinx older adults are at 1.5–2 times greater risk of clinical ADRD^2^ yet are more likely to have a missed or delayed diagnosis.^3^, ^4^ Minoritized individuals face unique stressors, such as interpersonal racial discrimination and structural racism, which can negatively impact health outcomes.^5^ Structural racism operates within interacting institutional forces to create a racial patterning of critical social determinants of health (SDOH),^6^ which are increasingly recognized as risk and protective factors for dementia.^7^ Ethnoracial differences in dementia risk are largely attributable to SDOH such as education and socioeconomic status, rather than genetic ancestry as there is no biological basis to race or ethnicity, which are social constructs.^8^, ^9^ Thus, understanding differences in ADRD risk requires contextualization of lived experiences and SDOH.

The accumulation of cerebral amyloid plaques is a pathological hallmark of AD that differs by race and ethnicity. In the original Imaging Dementia—Evidence for Amyloid Scanning (IDEAS) study, Black and Latinx participants with mild cognitive impairment (MCI) or dementia were significantly less likely than their White counterparts to have positive amyloid positron emission tomography (PET).^10^ This finding has now been replicated in various cohorts and across various amyloid biomarkers.^11^, ^12^, ^13^, ^14^, ^15^ This has significant implications both for clinical management, with novel anti‐amyloid therapies being a viable treatment option for patients with early‐stage clinical AD and confirmed AD pathology, as well as for clinical trials seeking to develop novel treatments. While there has been a push in recent years toward more inclusive practices in clinical trial recruitment for ADRD,^16^ Black and Latinx individuals are disproportionately excluded from these trials due to lack of amyloid positivity.^14^ To better understand disparities and move toward equity in early diagnosis of ADRD, it is essential to better understand the SDOH that drive these ethnoracial differences in amyloid PET positivity rates, providing necessary context for understanding ethnoracial disparities.

The New IDEAS study builds on the findings of the original IDEAS study^17^ seeking to characterize changes in patient management and health outcomes following amyloid PET in a more diverse and representative sample of cognitively impaired (MCI and dementia) older adults, and has found that nearly 60% of patients experienced a change in their management plan following amyloid PET.^18^ Secondary analysis of the original IDEAS study found ethnoracial differences in amyloid PET positivity,^10^ but was limited by a lack of inclusion of minoritized individuals (88% White), thus limiting the generalizability of findings. In the present analysis, we compare rates of amyloid PET positivity across ethnoracial groups and identify associations between various SDOH and amyloid PET positivity to better contextualize racial and ethnic differences in amyloid PET positivity in a highly diverse cohort of older adults with cognitive impairment.

## 2 METHODS

### 2.1 Study design

**RESEARCH IN CONTEXT**

1. **Systematic review**: The authors reviewed the literature using traditional (e.g., PubMed) sources and meeting abstracts and presentations. Recent studies have found ethnoracial differences in various Alzheimer's disease (AD) biomarkers, but the social determinants of health (SDOH)s driving these differences are not well understood. These relevant citations are appropriately cited.
2. **Interpretation**: We found significant differences in amyloid positron emission tomography (PET) positivity rates among ethnoracial groups and area deprivation categories with minoritized individuals having much lower rates and individuals in more distressed areas having higher rates. We did not find any of the SDOHs we measured to explain these differences.
3. **Future directions**: The cause of differing amyloid PET positivity rates across ethnoracial groups is still not well understood. Future work should include more comprehensive assessment of SDOH as well as examination of medical comorbidities and non‐AD neuropathologies to identify potential causes for these observed differences.

New IDEAS (NCT04426539) is an observational longitudinal cohort study seeking to evaluate the utility of amyloid PET in a diverse and representative sample of Medicare beneficiaries with cognitive impairment across the United States (US). This pragmatic study partnered with dementia specialists at clinical practices across the US to enroll cognitively impaired participants for amyloid PET. The study was conducted under Coverage with Evidence Development (CED) in partnership with the Centers for Medicare & Medicaid Services (CMS), which allowed for coverage of amyloid PET for Medicare beneficiaries to assess its clinical utility. More comprehensive details on study design, along with the full protocol and study materials, have been published elsewhere.^18^ The primary objective of New IDEAS was to identify changes in patient management and healthcare outcomes following amyloid PET in a large, diverse cohort of Medicare beneficiaries with cognitive impairment. The analyses presented in this manuscript focused on identifying associations between ethnoracial groups and amyloid PET positivity. Secondary analyses sought to understand associations between various SDOH and amyloid PET positivity and associations between ethnoracial groups and various SDOH and clinical variables.

The New IDEAS study was overseen by the American College of Radiology Center for Research and Innovation and a steering committee. A central institutional review board (Advarra) ensured compliance with ethical standards outlined in the 1964 Declaration of Helsinki and its amendments. Written informed consent was obtained by authorized site staff directly from the patient, or a legally authorized representative with patient assent for patients who lacked decisional capacity to provide informed consent. The template consent forms have been published elsewhere.^18^

### 2.2 Population

Dementia specialists enrolled patients who were receiving routine clinical care within their local clinical practices. To ensure a diverse and balanced cohort, the maximum enrollment number of participants by a single referring specialist was limited to 250 with rare single site exceptions. Inclusion criteria were: (1) Medicare beneficiary with Medicare as primary insurance; (2) meets clinical criteria for MCI or dementia;^19^ (3) structural brain imaging (magnetic resonance imaging [MRI] or computed tomography [CT]) within 24 months prior to enrollment; (4) clinical laboratory assessment within 12 months prior to enrollment; (5) ability to tolerate amyloid PET; (6) willing and able to provide consent (may be by proxy); and (7) neuropsychiatric syndrome can be classified into “clinically typical” or “clinically atypical” categories. Participants were excluded if they: (1) had normal cognition; (2) were at risk of significant psychological harm related to disclosure of amyloid PET results, as determined by dementia specialist; or (3) had previously learned of their amyloid or tau status. Additional exclusion criteria are outlined in the full study protocol.^18^

### 2.3 Study recruitment

The New IDEAS Study protocol pre‐specified that the study would enroll 7000 subjects in three groups: at least 2000 Black/African American (Black), at least 2000 Hispanic/Latinx (Latinx), and up to 3000 individuals from amongst all other racial and ethnic (AORE) groups. The multipronged recruitment strategy, which has been previously described,^18^ was spearheaded by Drs. Wilkins and Dilworth‐Anderson and intentionally used strategies proven in recruitment of Black and Latinx individuals leveraging resources from the Alzheimer's Association, the American College of Radiology, the Vanderbilt Recruitment Innovation Center,^20^, ^21^, ^22^, ^23^ and the UNC Center for Health Equity Research.^24^, ^25^ The recruitment strategy included a pause in enrollment of the AORE cohort while the study team addressed additional barriers to recruiting Black and Latinx individuals through enhanced community engagement, additional support for travel to PET imaging, expanded Spanish language resources, and on‐demand recruitment support for local sites.

### 2.4 Ascertainment of amyloid PET

All amyloid PET scans were performed at accredited imaging centers within 60 days of the referring physician completing the enrollment form using one of three US Food and Drug Administration (FDA)‐approved radiopharmaceuticals (florbetapir F 18, flutemetamol F 18, and florbetaben F 18) in accordance with established guidelines.^26^ Only facilities with full‐ring BGO, GSO, LSO or LYSO PET, PET/CT or PET/MRI scanners were eligible to participate; partial‐ring systems and dedicated NaI systems were not eligible for use in the New IDEAS Study. Imaging specialists performing visual interpretation of amyloid PET were required to be board‐certified in diagnostic radiology or nuclear medicine and were required to have completed vendor‐provided training courses specific to the amyloid imaging agent (or agents) used at their facility. Consistent with FDA guidelines, results were categorized as negative or positive, with negative results indicating sparse to no neuritic plaques and positive results indicating moderate to frequent neuritic plaques. Dementia specialists disclosed the results of amyloid PET to patients as part of routine clinical care.

### 2.5 Ascertainment of sociodemographic variables

Sociodemographic variables were collected at enrollment. Self‐identified race/ethnicity was based on at least one race category (American Indian or Alaskan Native, Asian or Asian American, Black or African American or African, Native Hawaiian or Pacific Islander, Middle Eastern or North African, White or European) and at least one ethnicity category (Hispanic or Latino or Spanish, not Hispanic or Latino or Spanish, not reported, or unknown). For these analyses, the Black group (*n* = 1248) was defined as anyone who identified as Black, African American, or African, with or without any other identity other than Latinx; the Latinx group (*n* = 1166) was defined as anyone who identified as Hispanic, Latinx, or Spanish, with or without any other identity other than Black. Participants who identified as both Black and Latinx (*n* = 5) were randomly assigned to either the Black (*n* = 2) or Latinx (*n* = 3) group. All other participants were placed in the AORE group (*n* = 3343); this group was predominantly individuals who identified as White without additional Black or Latinx ethnoracial identity (*n* = 3011). Gender identity was categorized as male or female. Transgender participants (*n* = 1) were assigned according to their gender identity. Educational attainment was collapsed for primary analyses, as follows: “Less than High School”, “Attended High School or an equivalent educational institution”, “Some college, Associate degree, or bachelor's degree”, and “Master's degree or higher”. Type of Medicare plan was defined as either traditional Medicare (fee for service) or Medicare Advantage. Area Deprivation Index (ADI; based on national scale), a scientifically validated measure of neighborhood‐level social disadvantage,^27^ was based on participants’ zip codes at time of study enrollment. Continuous ADI values were grouped by quintile into five categories: Prosperous (lowest ADI), Comfortable, Mid‐tier, At‐risk, and Distressed (highest ADI). Of note, this study emphasized recruitment in metro regions which tend to have a greater representation of individuals at the highest and lowest ADI values.

### 2.6 Assessment of cognition and medication use

Participants completed either the Montreal Cognitive Assessment (MoCA)^28^ or the Mini‐Mental State Examination (MMSE)^29^ as part of their standard clinical diagnostic evaluation within 3 months of study enrollment. For uniformity and ease of interpretation, MoCA scores were converted to equivalent MMSE scores based on previously published methods.^30^ Prior AD medication use was defined as the use of acetylcholinesterase inhibitors and/or memantine prior to amyloid PET scan.

### 2.7 Statistical analyses

Descriptive statistics are presented as median and interquartile range for continuous variables and percentage in each category for categorical variables. Differences in distribution of participant characteristics between ethnoracial groups were tested using chi‐square tests for categorical variables or analysis of variance (ANOVA) for continuous variables. The primary objective was to evaluate the association between ethnoracial groups and amyloid PET positivity status. To do so, we used a logistic regression generalized estimating equation (GEE) model with ethnoracial group as the only covariate and accounted for within site correlation using an exchangeable correlation structure. Secondary objectives included evaluating the association between various SDOHs and amyloid PET positivity and evaluating associations between various clinical and sociodemographic variables and ethnoracial group. Secondary analyses used a logistic regression GEE for binary outcomes and a linear regression GEE to compare MMSE scores between ethnoracial groups. Bonferroni correction was used to adjust for multiplicity for the two primary analyses, but no adjustments for multiple comparisons were made for secondary objectives. Multiple imputations with 20 imputations were used to handle missing data. In Supplementary Material, we provide results from and details on the analysis methods that adjust for covariates using g‐computation.^31^ The analysis followed a pre‐specified analysis plan that is available as part of the Supplementary Material. All statistical analyses were performed in R statistical package, version 4.4.3.

## 3 RESULTS

### 3.1 Participants

**FIGURE 1:**
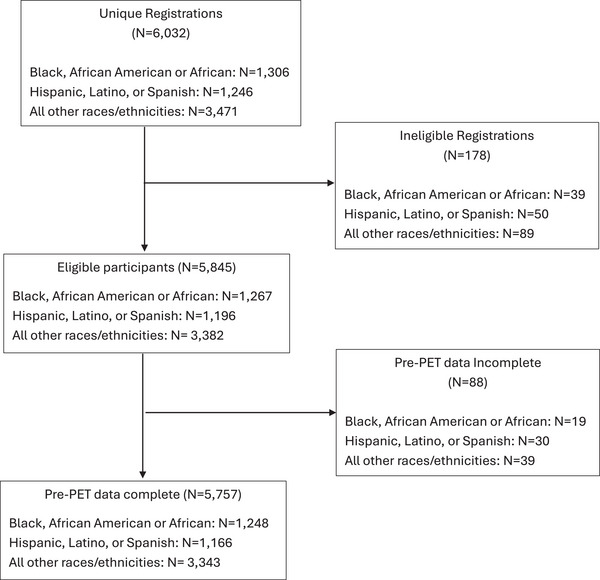
CONSORT diagram for this analysis, including the breakdown by ethnoracial group. CONSORT, Consolidated Standards of Reporting Trials.

A total of 6032 participants registered for the study between December 2020 and March 2024. The study was terminated for all participants prior to reaching enrollment targets on March 1, 2024 due to a number of reasons including retirement of the CMS National Coverage Determination on amyloid PET, termination of CED as a criterion for amyloid PET coverage on October 13, 2023, and increasing prior authorization denials, especially amongst Medicare Advantage beneficiaries. 206 participants did not complete informed consent or were later deemed ineligible, leaving 5757 participants in the final analyses with complete pre‐PET data see Figure 1. 1248 participants were Black, African American, or African (Black; 21.7%), 1166 participants were Hispanic, Latino, or Spanish (Latinx; 20.3%), and 3343 belonged to AORE group (58.1%). Median age at registration for the entire sample was 75 years (interquartile range [IQR]: 70–80) and median age at symptom onset was 72 years (IQR: 67–77). 3219 participants identified as female (55.9%). The majority of participants (*n* = 3606, 62.6%) were diagnosed with MCI as opposed to dementia (n = 2151, 37.4%). See Table 1 for participant characteristics overall and stratified by ethnoracial group.

**TABLE 1:** Participant characteristics stratified by ethnoracial group.

Parameter	Combined (*n* = 5757)	Black (*n* = 1248)	Latinx (*n* = 1166)	AORE (*n* = 3343)	*p‐value*	Black versus Latinx Adjusted *p*‐values	Black versus AORE Adjusted *p*‐values	Latinx versus AORE Adjusted *p*‐values
Age at registration, years	75 (70–80)	74 (68–79)	75 (70–80)	75 (71–80)	**<0.001**	**<0.001**	**<0.001**	0.50
Age at symptom onset, years	72 (67–77)	71 (66–76)	72 (67–77)	72 (67–77)	**0.001**	0.61	**0.001**	0.11
Gender, % female	55.9%	64.7%	62.1%	50.5%	**<0.001**	0.61	**<0.001**	**<0.001**
Diagnosis, dementia	37.4%	47.8%	44.5%	31.0%	**<0.001**	0.36	**<0.001**	**<0.001**
Equivalent MMSE score	25 (20–27)	22 (19–26)	22 (19–26)	26 (22–28)	**<0.001**	1.00	**<0.001**	**<0.001**
Pre‐PET AD medication usage, yes	46.0%	44.6%	44.9%	46.8%	0.31	1.00	0.60	0.85
Education attainment (Collapsed)					**<0.001**			
Less than high school	6.3%	3.5%	22.0%	1.9%		**<0.001**	**0.006**	**<0.001**
Attended high school or an equivalent educational institution	25.3%	32.8%	32.9%	19.9%		1.00	**<0.001**	**<0.001**
Some college, associate's degree, or bachelor's degree	46.9%	47.3%	35.5%	50.8%		**<0.001**	0.11	**<0.001**
Master's degree or higher	21.4%	16.4%	9.6%	27.4%		**<0.001**	**<0.001**	**<0.001**
ADI					**<0.001**			
Prosperous (1^st^ – 19^th^ percentile)	33.6%	23.4%	29.0%	39.0%		**0.006**	**<0.001**	**<0.001**
Comfortable (20^th^ – 39^th^ percentile)	25.3%	19.1%	26.8%	27.1%		**<0.001**	**<0.001**	1.00
Mid‐tier (40^th^ – 59^th^ percentile)	13.9%	16.1%	15.4%	12.6%		1.00	**0.008**	0.063
At‐risk (60^th^ – 79^th^ percentile)	8.3%	14.0%	9.9%	5.6%		**0.008**	**<0.001**	**<0.001**
Distressed (80^th^ – 100^th^ percentile)	5.9%	12.2%	5.3%	3.8%		**<0.001**	**<0.001**	0.096
Missing	12.9%	15.2%	13.6%	11.9%		0.88	**0.009**	0.38
Enrolled in Medicare Advantage	28.5%	40.6%	38.9%	20.3%	**<0.001**	1.00	**<0.001**	**<0.001**

*Note*: Values presented as median (interquartile range) for continuous variables and percentage for categorical variables. Bold font indicates *p*‐value < 0.05. The pairwise comparisons (Black versus Latinx, Black versus AORE and Latinx versus AORE) show the adjusted p‐values using the Bonferroni correction.

Abbreviations: AD, Alzheimer's disease; ADI, Area Deprivation Index; AORE, all other races/ethnicities; MMSE, Mini‐Mental State Examination; PET, positron emission tomography.

**FIGURE 2:**
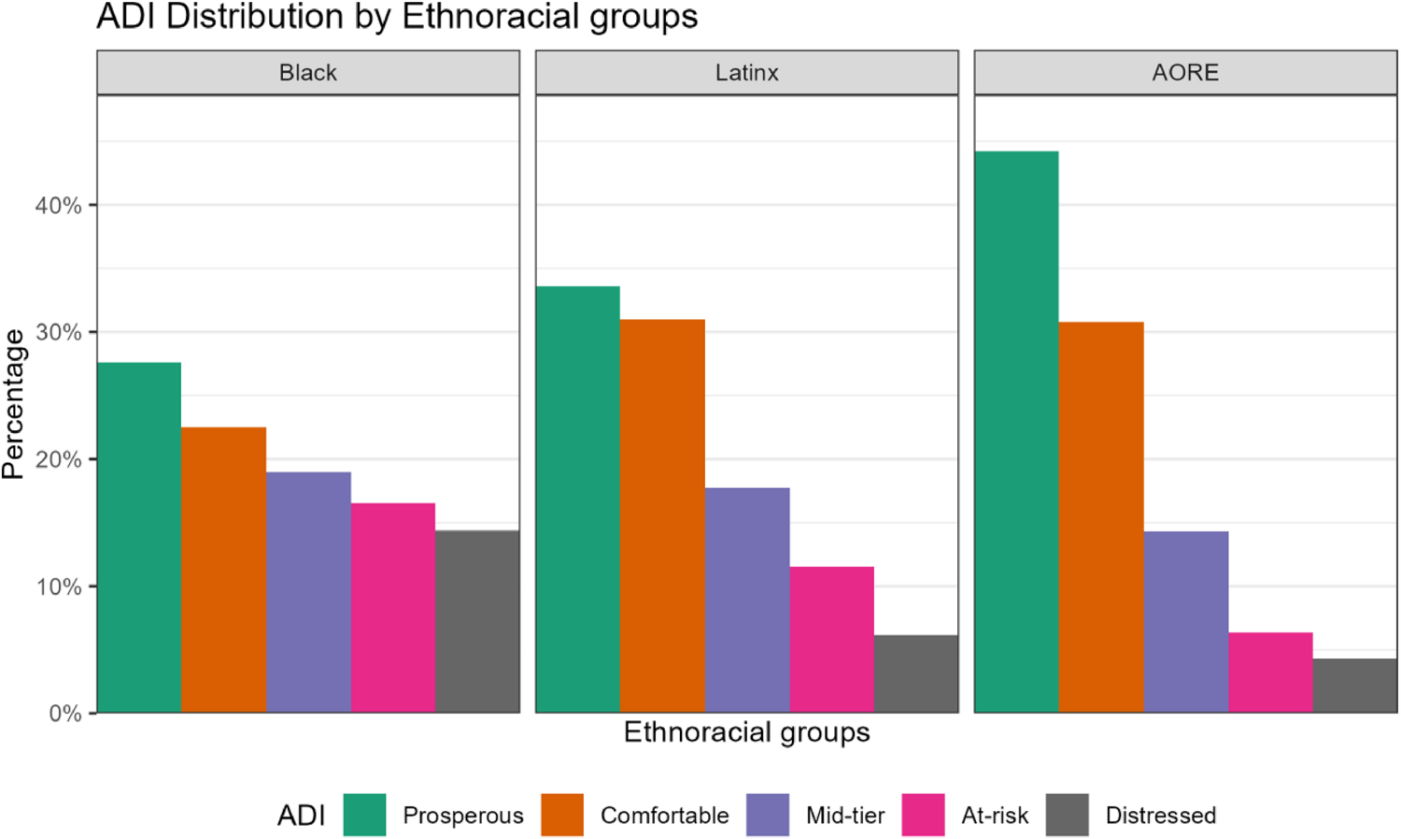
The ADI by ethnoracial group by ethnoracial group. Participants missing ADI were excluded. ADI, Area Deprivation Index.

MMSE scores differed across ethnoracial groups (*p* < 0.001), with Black participants (median = 22) and Latinx participants (median = 22) scoring lower than AORE group participants (median = 26; see Table 1). Latinx participants had the lowest levels of formal education, with 22% having no formal schooling or grade school education only compared to 3.5% of Black and 1.9% of AORE participants. AORE participants were more likely to have higher education (27.4% with a master's degree or above) compared to Black (16.4%) and Latinx (9.6%). There were notable differences in the location of high school by ethnoracial group, with Black participants predominantly attending urban schools (52.1%) while AORE participants were more likely to attend high school in a suburban setting (35.6%). Approximately one‐fourth of Latinx participants attended high school outside the United States. See Table 2 for a detailed breakdown of education characteristics by ethnoracial group. Medicare enrollment patterns differed (*p* < 0.001), with Black (40.6%) and Latinx (38.9%) participants having a higher representation of Medicare Advantage plans, while 80% of AORE participants were enrolled in traditional Medicare. Black (26.2%) and Latinx (15.2%) participants were more likely to be in the at‐risk/distressed ADI category than their AORE counterparts (9.4%; *p* < 0.001; see Figure 2). Pre‐PET ADRD‐related medication usage was similar across ethnoracial groups (*p* = 0.31). See Table 1 for participant characteristics stratified by ethnoracial group.

**TABLE 2:** Participant education characteristics by ethnoracial group.

Parameter	Combined (*n* = 5757)	Black (*n* = 1248)	Latinx (*n* = 1166)	AORE (*n* = 3343)
Education attainment				
No formal education	2.0%	0.4%	8.2%	0.5%
Grade school	4.3%	3.1%	13.7%	1.4%
Attended high school, did not graduate	3.3%	5.0%	6.9%	1.4%
High school graduate	18.2%	22.9%	20.9%	15.6%
High school equivalence	3.8%	4.9%	5.1%	2.9%
Some college or associate degree	24.4%	30.6%	20.5%	23.5%
Bachelor's degree	22.5%	16.7%	15.0%	27.3%
Master's degree	14.9%	12.7%	6.2%	18.7%
Doctoral or professional degree	6.6%	3.8%	3.4%	8.7%
Grade school attendance^a^				
Attended grade school all year	76.8%	79.5%	71.2%	93.6%
Often missed school during grade school	21.1%	20.5%	25.6%	6.4%
Missing	2.0%	0.0%	3.1%	0.0%
High school attendance (did not graduate)^b^				
Attended high school all year	70.4%	72.6%	61.3%	83.0%
Often missed school during high school	24.3%	24.2%	28.7%	17.0%
Missing	5.3%	3.2%	10.0%	0.0%
High school attendance (graduated)^c^				
Attended high school all year	91.0%	89.9%	88.5%	92.7%
Often missed school during high school	1.9%	0.7%	5.3%	1.0%
Missing	7.1%	9.4%	6.1%	6.3%
Location of high school attended^d^				
Urban (inner city)	36.3%	52.1%	36.7%	30.5%
Suburban	27.2%	14.9%	13.2%	35.6%
Rural	12.8%	15.3%	4.9%	14.2%
Outside US	8.6%	5.8%	25.6%	5.0%
Don't recall	2.2%	2.6%	2.2%	2.0%
Prefer not to answer	5.6%	6.2%	6.4%	5.2%
Missing	7.2%	3.2%	11.0%	7.7%
Type of high school attended****				
Public	78.8%	90.3%	67.3%	77.8%
Taught at home	0.1%	0.0%	0.2%	0.1%
Private	13.2%	5.8%	20.4%	13.9%
Missing	7.9%	3.9%	12.1%	8.3%

*Note*: Values denoted as percentages.

Abbreviation: AORE, all other races/ethnicities.

^a^ This question was only answered if participant's highest education was grade school.

^b^ This question was only answered if participant's highest level of education was attending high school but did not graduate.

^c^ This question was only answered if participant's highest level of education was graduating high school.

^d^ This question was only completed by participants who completed at least high school or higher level of education.

### 3.2 Differences in amyloid PET positivity by ethnoracial group

**FIGURE 3:**
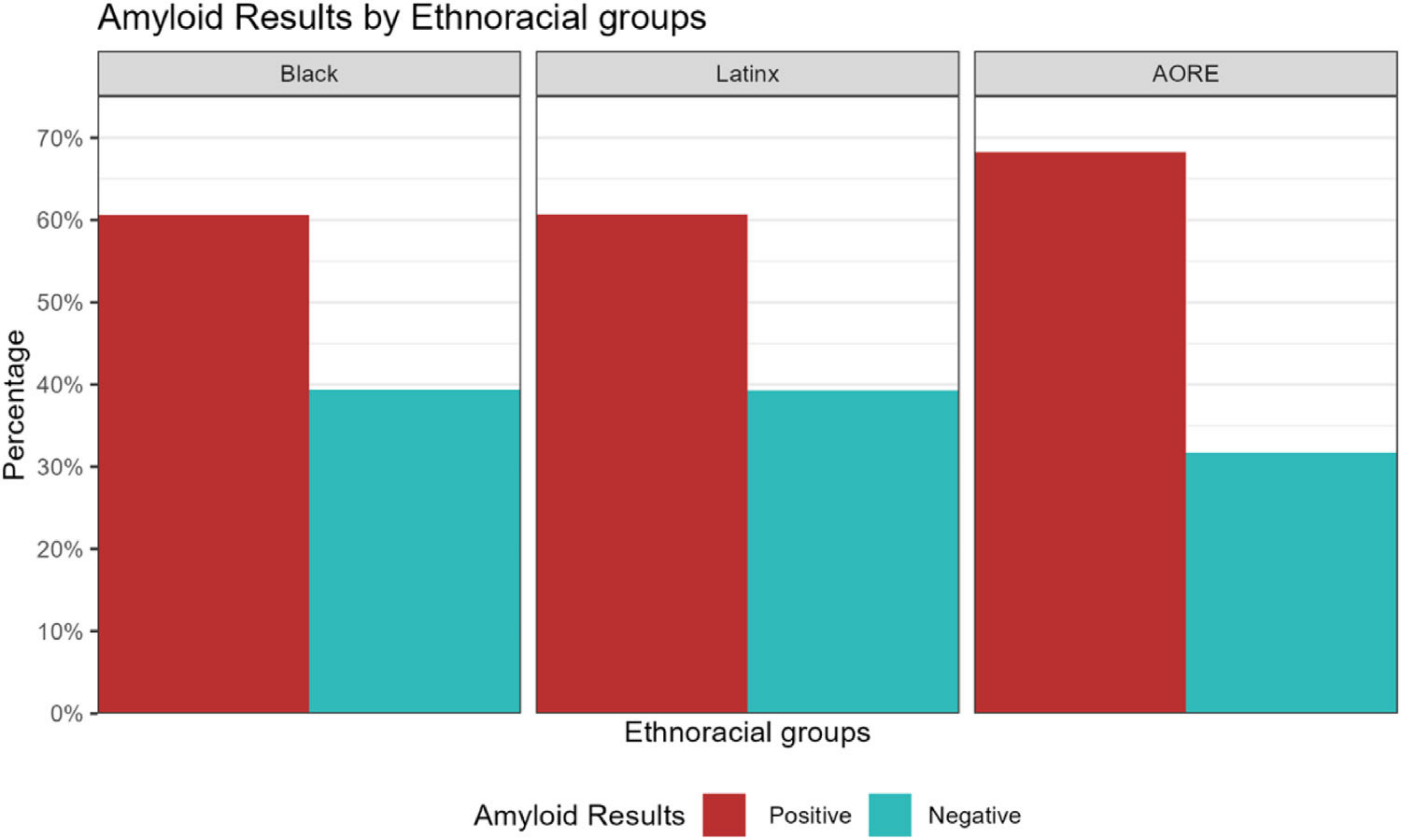
Amyloid results by ethnoracial group. Participants who did not complete an Amyloid PET scan were excluded from the figure. PET, positron emission tomography.

Results of the GEE logistic regression and fully adjusted models showing associations between ethnoracial group and amyloid PET positivity are shown in Table 3 and visually presented in Figure 3. AORE participants were more likely to have a positive amyloid PET result (60.0%) than those participants in the Black (50.1%) or Latinx (45.6%) groups. The logistic regression GEE models showed a significant difference in the rate of positive amyloid PET when comparing Black versus AORE participants (odds ratio [OR]: 0.72, 95% CI: 0.62–0.83) and when comparing Latinx versus AORE participants (OR: 0.78, 95% CI: 0.67‐0.91). These results persisted in fully adjusted models.

**TABLE 3:** Regression results for predicting amyloid PET positivity.

GEE logistic regression adjusting for enrollment site		
	OR	95% CI
**Ethnoracial group (reference = AORE)**		
Black	**0.72**	**0.62, 0.83**
Latinx	**0.78**	**0.67, 0.91**

Fully adjusted regression models*		
	G‐computation estimator	95% CI
**Ethnoracial group**		
Black	0.58	0.58, 0.58
Latinx	0.62	0.61, 0.62
AORE	0.69	0.69, 0.70

*Note*: *Models were adjusted for enrollment site, age at enrollment, age at symptom onset, equivalent MMSE score, gender, education, ADI, impairment level, medication use, and type of Medicare. G‐computation estimates probability of amyloid PET positivity for each group, adjusting for covariates. Bold font indicates *p*‐value < 0.05.

Abbreviations: AORE, all other races/ethnicities; CI, confidence interval; GEE, generalized estimating equations; OR, odds ratio; PET, positron emission tomography.

### 3.3 Differences in amyloid PET positivity by SDOH

The results of the four separate GEE logistic regression models showing associations between amyloid PET positivity and four categories of SDOH (gender, type of Medicare coverage, education attainment, and ADI), are shown in Table 4. All models adjusted for individual practice center. Compared to participants in the prosperous ADI group, participants in the comfortable (OR: 1.22, 95% CI: 1.05‐1.42) and distressed (OR: 1.40, 95% CI: 1.08–1.82) ADI groups were more likely to have a positive amyloid PET result. Individuals in the mid‐tier and at‐risk ADI groups did not have differing likelihood of amyloid PET positivity than those in the prosperous group. In fully adjusted models, amyloid PET positivity rates varied across ADI subgroups, with lowest risk for amyloid PET positivity in those in the prosperous group and highest risk in those in the distressed group see Table S1. Gender, type of Medicare coverage, and educational attainment did not show a statistically significant association with the likelihood of a positive amyloid PET result. In fully adjusted models, amyloid PET positivity rates differed across educational attainment groups; with increased rates at each increasing level of educational attainment see Table S1.

**TABLE 4:** GEE Logistic regression results for predicting amyloid PET positivity.

Parameter	OR	95% CI
**Gender (reference = female)**		
Male	0.99	0.88, 1.11
**Type of Medicare (reference = FFS)**		
Medicare Advantage	1.06	0.93, 1.21
**Education attainment (reference = some college, associate's degree, bachelor's degree)**		
<HS	1.25	0.96, 1.64
Attended HS or HS equivalent	1.22	0.95, 1.58
Master's degree or higher	1.23	0.93, 1.61
**ADI (reference = prosperous)**		
Comfortable	**1.22**	**1.05, 1.42**
Mid‐tier	1.18	0.99, 1.41
At‐risk	1.11	0.88, 1.40
Distressed	**1.40**	**1.08, 1.82**

*Note*: Models were adjusted for enrollment site. Bold font indicates *p*‐value < 0.05.

Abbreviations: ADI, Area Deprivation Index; CI, confidence interval; FFS, fee‐for‐service; GEE, generalized estimating equations; HS, high school; OR, odds ratio; PET, positron emission tomography.

### 3.4 Differences in clinical outcomes by ethnoracial group

The results of the GEE logistic regression models show associations between ethnoracial groups and likelihood of dementia diagnosis, ADRD medication use prior to amyloid PET, and type of Medicare enrollment, are shown in Table 5. All models adjusted for individual practice center. Black and Latinx participants were more likely to have a diagnosis of dementia (Black participants’ OR: 2.09, 95% CI: 1.83–2.40; Latinx participants’ OR: 1.89, 95% CI: 1.64–2.17) than AORE participants. Similarly, Black (OR: 2.71, 95% CI 2.36‐3.12) and Latinx (OR: 2.52, 95% CI: 2.17–2.91) participants were more likely than AORE participants to be enrolled in a Medicare Advantage plan. Compared to the original IDEAS cohort, rates of Medicare Advantage enrollment were significantly higher across all racial groups see Table S2. There were no significant ethnoracial differences in ADRD medication use prior to amyloid PET. In fully adjusted models, ethnoracial differences were noted in dementia diagnosis Table S3, AD medication usage prior to PET Table S4, and Medicare Advantage enrollment Table S5, with Black and Latinx participants having an increased likelihood of dementia and Medicare Advantage enrollment, and lower likelihood of AD medication use prior to PET than AORE participants.

**TABLE 5:** Associations of ethnoracial groups with secondary outcomes.

Parameter	OR	95% CI
**Diagnosis of dementia (vs. MCI; reference = AORE)**		
Black	**2.09**	**1.83, 2.40**
Latinx	**1.89**	**1.64, 2.17**
**AD medication use prior to PET (vs. no medications; reference = AORE)**		
Black	0.92	0.81, 1.05
Latinx	0.91	0.80, 1.05
**Medicare Advantage (vs. FFS; reference = AORE)**		
Black	**2.71**	**2.36, 3.12**
Latinx	**2.52**	**2.17, 2.91**

*Note*: Models were adjusted for enrollment site. Bold font indicates *p*‐value < 0.05.

Abbreviations: AD, Alzheimer's disease; AORE, all other races/ethnicities; CI, confidence interval; FFS, fee‐for‐service; MCI, mild cognitive impairment; OR, odds ratio; PET, positron emission tomography.

Linear regression GEE models relating ethnoracial group to MMSE scores, adjusting for individual practice center, showed that Black (β€ = ‐2.85, 95% CI: ‐3.19 –2.51) and Latinx (β€ = ‐2.91, 95% CI: ‐3.26 –2.56) participants had lower average MMSE scores than AORE participants. These results persisted in fully adjusted models Table S6.

## 4 DISCUSSION

In this large, multisite cohort study of cognitively impaired Medicare beneficiaries from across the United States, we found strong evidence for ethnoracial differences in amyloid PET positivity rates, with Black and Latinx participants having significantly lower odds of a positive amyloid PET than their AORE counterparts. ADI was associated with amyloid PET positivity, with individuals in the most distressed ADI category having the highest likelihood of amyloid PET positivity, while social factors such as gender, type of Medicare plan, or educational attainment, were not. Ethnoracial group was also a significant predictor of several secondary outcomes, with Black and Latinx participants more likely than AORE participants to be enrolled in Medicare Advantage, have a dementia diagnosis at enrollment, score lower on cognitive tests, and in adjusted models, be less likely to use AD medications prior to amyloid PET. These findings expand existing literature on ethnoracial differences in AD biomarkers, highlighting key SDOH that contextualize these disparities and provide targets for future investigation and intervention.

Secondary analysis of data from the original IDEAS study showed differences in amyloid PET positivity rates across ethnoracial groups yet lacked diversity (88% White individuals).^10^ These findings have been replicated elsewhere, such as in the Anti‐Amyloid in Asymptomatic AD (A4) study, with similar limitations (96% White).^32^ In the New IDEAS study, we increased representation of minoritized groups including Black (22%) and Latinx (20%), enabling replication of our past findings in a more diverse cohort and deeper characterization of SDOH. In the context of novel anti‐amyloid treatments and clinical trials focusing heavily on AD dementia, our findings have profound importance for equitable care. Fewer Black and Latinx patients will qualify for anti‐amyloid treatments, thus limiting the potential societal benefit of these new therapies. Further, these groups are less likely to meet eligibility requirements for clinical trials, further restricting advancements in effective treatments for diverse populations. Indeed, this is already the case, as underrepresentation in recent AD clinical trials is due largely to disproportionate study exclusions of minoritized individuals not showing biomarker positivity.^14^ This has also been shown to be true in AD drug trials in asymptomatic participants as well.^12^ Taken together, the current landscape of treatment and drug development for cognitive impairment will further exacerbate existing ethnoracial disparities in dementia, underscoring the need for developing treatments for non‐AD causes of cognitive impairment.

Our findings of lower amyloid positivity rates in Black and Latinx older adults with cognitive impairment suggest a higher prevalence of non‐AD etiologies in these populations. Although AD is the most common cause of dementia, non‐amyloid dementias, such as vascular dementia, are also prevalent, particularly in Black and Latinx populations.^33^ Vascular risk factors such as hypertension and diabetes are more common in Black and Latinx American individuals compared to non‐Hispanic White adults,^34^ potentially explaining differing rates of cerebrovascular disease in these groups. This suggests that amyloid‐targeted interventions alone will be less effective at treating cognitive impairment in these groups while alternative interventions, such as lifestyle interventions, may be necessary. Recent findings from the US POINTER study suggest that a structured lifestyle intervention can improve cognitive function similarly across ethnoracial groups.^35^ Our results emphasize the potential benefit of non‐amyloid interventions for the treatment and prevention of cognitive impairment.

Despite lower rates of amyloid positivity, Black and Latinx participants were more likely to be diagnosed with dementia compared to AORE participants. These findings align with past research showing minoritized individuals are less likely to receive an MCI diagnosis,^36^ and more likely to present for care at the dementia stage.^37^ In resource‐deprived areas, only the most impaired individuals are likely to access medical care while those with milder deficits often go undiagnosed. This may have caused selection bias toward worse cognitive impairment in these groups, as Black and Latinx groups had a higher percentage of individuals in Distressed and At‐Risk ADI groups. Indeed, delayed or missed diagnosis is more common in areas with lower neighborhood socioeconomic status^38^ and among Black American individuals with lower incomes.^4^ Notably, when diagnostic resources are provided, (e.g., in observational studies), Black and Latinx individuals have *higher* rates of MCI than AORE participants.^39^ These findings have important clinical implications, as amyloid‐targeting therapies are more effective at earlier stages, a trend likely to persist for new ADRD treatments.

We found that Black and Latinx participants scored lower on cognitive screening measures than AORE individuals, an oft reported finding in the field. Widely used measures like the MMSE and MoCA show significant differences across ethnoracial groups with minoritized individuals scoring lower on average.^40^, ^41^ These differences likely arise because the tests were developed in predominantly Non‐Hispanic White populations with different sociodemographic/cultural contexts from Black and Latinx communities.^42^, ^43^ Missed/delayed diagnosis can lead to treatment delays, but strict thresholds for cognitive test scores may lead to over‐diagnosis and exclusion from clinical trials, even in those minoritized individuals who *are* amyloid positive. The CLARITY‐AD trial, which demonstrated lecanemab's efficacy, required MMSE scores ≥ 22,^44^ the median MMSE score for minoritized individuals in this study. Prevention trials targeting asymptomatic biomarker‐positive individuals have even stricter requirements (e.g., ≥ 27).^45^

Beyond clinical barriers to effective treatment of minoritized populations with cognitive impairment, we found salient evidence of systemic barriers to care. Black individuals had 2.71 times higher odds, and Latinx individuals had 2.52 times higher odds, of being enrolled in a Medicare Advantage plan, compared to AORE. This phenomenon has been increasing over time.^46^, ^47^ Compared to the original IDEAS study, enrollment in Medicare Advantage plans in New IDEAS more than doubled. While Medicare Advantage plans vary in quality, plans selected by ethnoracial minority beneficiaries are often associated with poorer health outcomes, including less preventive care, fewer specialty referrals, and reduced follow‐up after hospitalizations.^47^, ^48^ Minoritized populations generally have more Medicare Advantage plans available, but fewer high‐rated plans than AORE individuals.^49^ Consequently, Black and Latinx individuals are more likely to enroll in plans restricting access to care. Anecdotally, we found notable difficulties in obtaining successful coverage for amyloid PET in Medicare Advantage plans, with many plans denying or delaying authorization despite a CED approval letter.

Despite significant ethnoracial differences in many SDOH examined, we did not find gender, type of Medicare plan, or educational attainment to be associated with amyloid PET positivity rates. Although SDOH are known to influence health outcomes,^9^ the components examined here did not explain ethnoracial differences in amyloid positivity. This may reflect study limitations including missing data and a lack of life course or individual‐level data. In the original IDEAS study, modest sex differences in amyloid positivity were observed, driven by higher rates in Black women compared to Black men.^50^ The absence of replication here may stem from sample size differences, leaving the present study underpowered to detect this relatively small effect. Similarly, past studies have linked education with amyloid deposition,^51^, ^52^, ^53^ which was not replicated here. These earlier studies were rather small, with a 2025 meta‐analysis including just 288 participants.^53^ Our study is much larger, more ethnoracially diverse, and includes a larger proportion of individuals with very low educational levels.

We found that individuals in the lowest ADI group (most favorable socioeconomic conditions) were more likely to be amyloid positive, likely due to overrepresentation of AORE individuals. Although Black and Latinx groups had greater representation in the highest ADI groups, individuals with the highest ADI scores were also more likely to be amyloid positive, consistent with past work linking higher ADI to AD neuropathology at autopsy.^54^ Importantly, there are significant ethnoracial differences in brain donation,^55^ and this past work did not report racial or ethnic breakdown but did report fewer participants from the most deprived areas. Our results extend this work by including a diverse sample with representation across ADI groups.

This study has numerous strengths. New IDEAS is, to our knowledge, the largest study examining ethnoracial differences in amyloid PET positivity. Our high representation of individuals from lower educational strata also is unique amongst AD/ADRD studies. Beyond describing differences in amyloid PET positivity, we acquired detailed information on SDOH and analyzed their relation to amyloid PET status. This study expands our past findings by replicating them in a more diverse sample and offering insights into social factors driving observed differences. This study also had some important limitations, including potential selection bias, missing data (addressed via multiple imputation), and a focus on current rather than lifetime ADI. We were pragmatically limited in our ability to consider individuals of multiple ethnoracial identities, and while the number of these individuals was low enough to be unlikely to affect results, we are unable to generalize these findings to individuals of multiple ethnoracial identities. Additionally, all participants had access to specialists, likely excluding the most disadvantaged people. The study's pragmatic design is in the context of a healthcare system that limits access, increases barriers to care, and often overlooks minoritized individuals. This was evident in numerous facets of the study, including recruitment, physician diagnosis, and insurance coverage for amyloid PET. Finally, important genetic factors, such as *apolipoprotein E (APOE)* genotype, which may vary across ethnoracial groups, were not considered due to pragmatic concerns regarding participant accrual. However, *APOE* genotyping was later completed for many participants, and we hope to explore how genetic factors influence amyloid PET positivity rates in a future manuscript.

The clinical landscape of dementia has been transformed with the development of anti‐amyloid treatments and amyloid biomarkers. These advancements usher in a new era of care marked by early diagnosis, effective treatments, and potentially even prevention of cognitive impairment. However, like so many clinical and scientific advancements of the past, these breakthroughs will not have an equal impact on all people and will instead likely further increase existing disparities. Our findings highlight the need to develop diagnostic tools and treatments for dementias to ensure equitable care is available to all people with cognitive impairment.

## CONFLICT OF INTEREST STATEMENT

C.J.B. reports research support from the National Institute on Aging and the Alzheimer's Association. J.S., L.H., M.T., I.G., and C.G. report research support from the Alzheimer's Association and the American College of Radiology. K.S. was supported by the American College of Radiology through institutional funding. C.G. and BEH report support from the American College of Radiology. C.C.W. reports receiving grants from the Alzheimer's Association and the NIA. He has received honorariums from LCN, Kinetix Group, and Onviv Inc. MCC is a full‐time employee of the Alzheimer's Association. E.G. and A.M. are salaried employees of the American College of Radiology. M.B.H. reports research support from the National Institute on Aging and the National Institute for Neurological Disorders and Stroke. R.A.R. reports research support from the National Institute on Aging and the Alzheimer's Association and is a consultant for Amydis Inc, Bioivt, Lexeo, Keystone Bio, Allyx, DiamiR, Ionis, and PrecisionMed. B.A.S. reports receiving grants to institution from the American College of Radiology (ACR) during the conduct of the study; personal fees from Avid Radiopharmaceuticals, Curium Pharma, Progenics Pharmaceuticals, Lantheus Medical Imaging, the American Medical Foundation for Peer Review and Education, Siemens Healthineers (for spouse), ACR (also for spouse), Capella Imaging, ECOG‐ACRIN Medical Research Foundation (also for spouse), Evicore Healthcare (for spouse), GE Healthcare, and Radiological Society of North America (also for spouse) outside the submitted work; and grants from Curium Pharma, Progenics Pharmaceuticals, ImaginAb, and Blue Earth Diagnostics outside the submitted work. R.A.W. reports grants from the NIH and NIA and consulting fees from Genentech PanNeuro. She is the epidemiology section editor for Alzheimer's and Dementia. C.J.W. is a full‐time employee of the Alzheimer's Association. G.D.R. received research support from Eli Lilly, GE Healthcare and Life Molecular Imaging for New IDEAS, and additional research support from Genentech. He has served as a paid consultant for Bristol Myers Squibb, C2N, Eli Lilly, Johnson & Johnson, Merck, Novo Norodisk, and Roche. He is an Associate Editor for JAMA. C.H.W. reports grants from the NIH, PCORI, and the American College of Radiology. P.D.A. reports no conflicts of interest relevant to this work. Author disclosures are available in the supporting information. Author disclosures are available in the supporting information.

## CONSENT STATEMENT

Authorized site staff obtained written informed consent directly from the patient or, in cases in which the patient lacked capacity to consent, from a legally authorized representative with patient for all human subject participants in this study.

## Supporting information



Supporting Information

Supporting Information
