# Supplementary material for: Differences in amyloid PET positivity based on ethnoracial group and social determinants of health: The new IDEAS study
Source: Alzheimers Dement. 2026 May 27;22(5):e71406. doi: 10.1002/alz.71406 (PMC13240095; doi:10.1002/alz.71406)
Supplement: Supplementary file 2 — Supporting Information [file ALZ-22-e71406-s001.docx]

**Supplementary Material for Differences in Amyloid PET Positivity Based on Ethnoracial Group and Social Determinants of Health: The New IDEAS Study**

Details on statistical methods for adjusted models: Additional analysis accounting for covariates: To account for the effect of other covariates we used the g-formula with a logistic and linear regression models (results presented in appendix). The non-parametric bootstrap was used for variance estimation using 200 bootstrap samples with 5 multiple imputations implemented with the bootimpute package in R.^2^ The regression model included age at registration, age of symptoms, individual practice center, equivalent MMSE score, gender, education attainment, cognitive impairment, Medicare, ADI, and AD medication use prior to PET. If participants are missing age of symptoms, then age at registration was used. The analog analysis was done to examine associations with impairment levels, AD medication use prior to PET, type of medicare plan and MMSE as the outcome.

| **Supplemental Table 1. Fully Adjusted Regression Results for Predicting Amyloid PET Positivity** | | |
| --- | --- | --- |
|  | **G-computation Estimator** | **95% CI** |
| **Ethnoracial Group** |  |  |
| Black | 0.58 | 0.58, 0.58 |
| Latinx | 0.62 | 0.61, 0.62 |
| AORE | 0.69 | 0.69, 0.70 |
| **Education Attainment** |  |  |
| <High school | 0.57 | 0.57, 0.57 |
| Attended HS or HS equivalent | 0.65 | 0.65, 0.65 |
| Some college, associate’s degree, bachelor’s degree | 0.66 | 0.66, 0.67 |
| Master’s degree or higher | 0.66 | 0.66, 0.66 |
| **Area Deprivation Index** |  |  |
| Prosperous | 0.63 | 0.63, 0.63 |
| Comfortable | 0.67 | 0.67, 0.67 |
| Mid-tier | 0.67 | 0.67, 0.67 |
| At-risk | 0.66 | 0.66, 0.66 |
| Distressed | 0.71 | 0.71, 0.72 |
| Note. Models were adjusted for enrollment site, age at enrollment, age at symptom onset, equivalent MMSE score, gender, education, ADI, impairment level, medication use, and type of Medicare. G-computation estimate probability of amyloid PET positivity for each group, adjusting for covariates. Black, Black, African American, or African; CI, confidence interval; HS, high school; Latinx, Hispanic, Latino, or Spanish; AORE, Neither Black nor LatinX; PET, positron emission tomography. | | |

**Supplemental Table 2. Comparison of Medicare Advantage Use in IDEAS and New IDEAS**

| **Type of Medicare Beneficiary** | **IDEAS**  ***n***  ***(column %)*** | **New IDEAS**  ***n***  ***(column %)*** | **Chi Square Test of Equality of Proportions**  ***(p-value)*** | |
| --- | --- | --- | --- | --- |
| **All Participants Included in Analysis** | | | | |
| Fee for Service | 15,744 *(86.24)* | 4,119 *(71.65)* | <0.0001 | |
| Medicare Advantage | 2,513 *(13.76)* | 1,638 *(28.45)* |  |  |
| **Black/African American/African** | | | | |
| Fee for Service | 512 *(79.75)* | 741 *(59.38)* | <0.0001 | |
| Medicare Advantage | 130 *(20.25)* | 507 *(40.63)* |  |  |
| **Hispanic/Latinx** | | | | |
| Fee for Service | 669 *(80.80)* | 712 *(61.06)* | <0.0001 | |
| Medicare Advantage | 159 *(19.20)* | 454 *(38.94)* |  |  |
| **All Other Races/Ethnicities** | | | | |
| Fee for Service | 14,563 *(86.75)* | 2,666 *(79.75)* | <0.0001 | |
| Medicare Advantage | 2,224 *(13.25)* | 677 *(20.25)* |  |  |
| **Note.** IDEAS and New IDEAS had different categories for race, with IDEAS collecting ethnicity as a separate question from race. Since both studies allowed participants to identify more than one race, participants could identify themselves in the same manner in both studies. Nine cases in IDEAS were both Black and Hispanic. These were randomly assigned to one or the other group using a uniform random value generated by SAS. In New IDEAS, cases in both categories were randomly assigned to one group using block randomization. | | | |  |

| **Supplemental Table 3. Fully adjusted regression results for predicting dementia diagnosis** | | |
| --- | --- | --- |
|  | **G-computation Estimator** | **95% CI** |
| **Education Attainment** |  |  |
| <High school | 0.39 | 0.39, 0.40 |
| Attended HS or HS equivalent | 0.38 | 0.38, 0.38 |
| Some college, associate’s degree, bachelor’s degree | 0.37 | 0.37, 0.37 |
| Master’s degree or higher | 0.36 | 0.36, 0.36 |
| **Area Deprivation Index** |  |  |
| Prosperous | 0.36 | 0.35, 0.36 |
| Comfortable | 0.37 | 0.37, 0.37 |
| Mid-tier | 0.38 | 0.38, 0.38 |
| At-risk | 0.42 | 0.42, 0.42 |
| Distressed | 0.41 | 0.41, 0.41 |
| Note. Models were adjusted for enrollment site, age at enrollment, age at symptom onset, equivalent MMSE score, gender, education, ADI, impairment level, medication use, and type of Medicare. G-computation estimate the probability of dementia diagnosis for each group, adjusting for covariates. CI, confidence interval; HS, high school. | | |

| **Supplemental Table 4. Fully adjusted regression results for predicting AD medication usage prior to Amyloid PET** | | |
| --- | --- | --- |
|  | **G-computation Estimator** | **95% CI** |
| **Ethnoracial Group** |  |  |
| Black | 0.41 | 0.41, 0.41 |
| Latinx | 0.42 | 0.42, 0.42 |
| AORE | 0.49 | 0.49, 0.49 |
| **Education Attainment** |  |  |
| <High school | 0.40 | 0.40, 0.40 |
| Attended HS or HS equivalent | 0.46 | 0.46, 0.46 |
| Some college, associate’s degree, bachelor’s degree | 0.46 | 0.46, 0.46 |
| Master’s degree or higher | 0.48 | 0.48, 0.48 |
| **Area Deprivation Index** |  |  |
| Prosperous | 0.41 | 0.41, 0.42 |
| Comfortable | 0.47 | 0.47, 0.47 |
| Mid-tier | 0.51 | 0.51, 0.51 |
| At-risk | 0.51 | 0.51, 0.51 |
| Distressed | 0.50 | 0.50, 0.50 |
| Note. Models were adjusted for enrollment site, age at enrollment, age at symptom onset, equivalent MMSE score, gender, education, ADI, impairment level, medication use, and type of Medicare. G-computation estimate the probability of AD medication usage prior to amyloid PET for each group, adjusting for covariates. AD, Alzheimer’s disease; ADI, Area Deprivation Index; AORE, all other races/ethnicities; CI, confidence interval; HS, high school; PET, positron emission tomography. | | |

| **Supplemental Table 5. Fully adjusted regression results for predicting Medicare Advantage enrollment** | | |
| --- | --- | --- |
|  | **G-computation Estimator** | **95% CI** |
| **Ethnoracial Group** |  |  |
| Black | 0.38 | 0.38, 0.38 |
| Latinx | 0.38 | 0.38, 0.38 |
| AORE | 0.21 | 0.21, 0.21 |
| **Education Attainment** |  |  |
| <High school | 0.30 | 0.30, 0.30 |
| Attended HS or HS equivalent | 0.29 | 0.29, 0.30 |
| Some college, associate’s degree, bachelor’s degree | 0.28 | 0.28, 0.28 |
| Master’s degree or higher | 0.27 | 0.27, 0.27 |
| **Area Deprivation Index** |  |  |
| Prosperous | 0.25 | 0.25, 0.25 |
| Comfortable | 0.27 | 0.27, 0.27 |
| Mid-tier | 0.32 | 0.32, 0.32 |
| At-risk | 0.33 | 0.33, 0.33 |
| Distressed | 0.38 | 0.38, 0.38 |
| Note. Models were adjusted for enrollment site, age at enrollment, age at symptom onset, equivalent MMSE score, gender, education, ADI, impairment level, medication use, and type of Medicare. G-computation estimates the probability of Medicare Advantage enrollment for each group, adjusting for covariates. AORE, all other races/ethnicities; CI, confidence interval; HS, high school; PET, positron emission tomography. | | |

| **Supplemental Table 6. Fully adjusted regression results for predicting MMSE score** | | |  |
| --- | --- | --- | --- |
|  | **G-computation Estimator** | **95% CI** | |
| **Ethnoracial Group** |  |  | |
| Black | 22.03 | 22.02, 22.04 | |
| Latinx | 22.68 | 22.67, 22.69 | |
| AORE | 23.90 | 23.90, 23.91 | |
| **Education Attainment** |  |  | |
| <High school | 20.01 | 19.99, 20.03 | |
| Attended HS or HS equivalent | 22.43 | 22.42, 22.44 | |
| Some college, associate’s degree, bachelor’s degree | 23.62 | 23.61, 23.63 | |
| Master’s degree or higher | 24.36 | 24.36, 24.37 | |
| **Area Deprivation Index** |  |  | |
| Prosperous | 22.83 | 22.82, 22.84 | |
| Comfortable | 23.29 | 23.28, 23.30 | |
| Mid-tier | 23.71 | 23.70, 23.72 | |
| At-risk | 23.68 | 23.67, 23.69 | |
| Distressed | 23.79 | 23.78, 23.81 | |
| Note. Models were adjusted for enrollment site, age at enrollment, age at symptom onset, equivalent MMSE score, gender, education, ADI, impairment level, medication use, and type of Medicare. G-computation estimates mean MMSE scores, adjusting for covariates. ADI, Area Deprivation Index; AORE, all other races/ethnicities; CI, confidence interval; HS, high school; MMSE, Mini-Mental State Examination; PET, positron emission tomography. | | |  |
